# *Allium pallasii* and *A. caricifolium*—Surprisingly Diverse Old Steppe Species, Showing a Clear Geographical Barrier in the Area of Lake Zaysan

**DOI:** 10.3390/plants11111465

**Published:** 2022-05-30

**Authors:** Nikolai Friesen, Lisa Grützmacher, Mikhail Skaptsov, Polina Vesselova, Vladimir Dorofeyev, Alexander N. Luferov, Nazgul Turdumatova, Georgii Lazkov, Sergei V. Smirnov, Alexander I. Shmakov, Herbert Hurka

**Affiliations:** 1 Botanical Garden, University of Osnabrück, Albrechtstrasse 29, 49076 Osnabrück, Germany; lgruetzmache@uni-osnabrueck.de (L.G.); herbert.hurka@osnanet.de (H.H.); 2 Department of Pharmacy and Natural Sciences, A.P. Nelyubin Institute of Pharmacy, I.M. Sechenov First Moscow State Medical University, Ministry of Health of the Russian Federation, Izmailovsky Boulevard 8, 105043 Moscow, Russia; luferovc@mail.ru; 3 South Siberian Botanical Garden, Altai State University, Lenina Str. 61, 656049 Barnaul, Russia; mr.skaptsov@mail.ru (M.S.); serg_sm_@mail.ru (S.V.S.); alex_shmakov@mail.ru (A.I.S.); 4 Institute of Botany and Phytointroduction of the Committee of Forestry and Wildlife, Ministry of Ecology, Geology and Natural Resources of the Republic of Kazakhstan, 480070 Almaty, Kazakhstan; pol_ves@mail.ru; 5 Komarov Botanical Institute of RAS, Professor Popov Str. 2, 197376 Saint Petersburg, Russia; vdorofeyev@yandex.ru; 6 Institute of Biology, National Academy of Sciences, Bishkek 720071, Kyrgyzstan; nazgul.turdumatova@gmail.com (N.T.); glazkov1963@mail.ru (G.L.)

**Keywords:** vicariant species, steppe evolution, internal transcribed spacer, plastid DNA, Zaysan Lake

## Abstract

Polymorph *Allium pallasii* s.l. from monotypic *A.* sect. *Pallasia* was studied using a wide spectrum of methods and divided into two clearly morphologically, geographically, cytologically and genetically isolated species: *A. pallasii* s. str.—North-East Kazakhstan, Western Siberia, and the Altai Mountains; *A. caricifolium*—Kyrgyzstan, Northwest China, South-East Kazakhstan until Zaysan Lake in the east. Despite serious genetic differences, both species are sisters and are related to species of the *A.* sect. *Codonoprasum* (Subg. *Allium*). *Allium caricifolium* differs from *A. pallasii* s. str. by taller stems, dense inflorescence, and with filaments longer than perianth. The possible phylogenetic reasons for the separation of these species are discussed. A nomenclature analysis of synonyms was carried out.

## 1. Introduction

*Allium* L. (Amaryllidaceae J.St.-Hil.: Allioideae Herb.) is one of the largest monocot genera with more than 1000 species [1] naturally distributed throughout the northern hemisphere [2,3,4,5,6,7]. The main centres of biodiversity are located in arid and sub-arid regions of Southwestern and Central Asia, and in the Mediterranean region. The significantly smaller centre is in western North America [5,7,8,9]). The genus is characterized by bulbs (often formed on rhizomes) enclosed in membranous, fibrous, or reticulate tunics, free or basally connate sepals, and usually a subgynobasic style [7]. The overwhelming morphological diversity in the genus is mirrored by a complicated taxonomic structure consisting of 15 subgenera and 72 sections of three evolutionary lineages [4,7].

All subsequent phylogenetic studies [7,9,10,11,12,13,14,15] confirmed the division of *Allium* into three major evolutionary lineages with the monophyletic origin of all subgenera included in the first and second evolutionary lineages. The phylogenetic relationships in the youngest third lineage are less clear. According to the latest studies, many subgenera are not monophyletic in the third evolution line. This mainly affects the subgenera *Cepa* (Mill.) Radić, *Reticulatobulbosa* (Kamelin) N.Friesen, *Rhizirideum* G.Don ex Koch) Wendelbo, *Polyprason* Radić and possible *Allium* [5,9,12,13,16].

*Allium pallasii* Murrey [17], the single representative of the monotypic *A.* sect. *Pallasia* (Tsag.) F.O. Khas., R.M. Fritsch & N. Friesen (subgen. *Allium*), is widespread in the steppe from central Kazakhstan in the west and to the Kulunda Steppe in West Siberia, Russia in the east and from 54° N in the north in Kazakhstan to Kyrgyzstan, south-east Uzbekistan, and north-west China in the south. There are two isolated occurrences of *A. pallasii* in the Kurai Steppe in the Altai Mountains and the Dzungarian Gobi in East Mongolia [18,19,20]. From the beginning, we were interested in the relationship of plants from Altai with the *A. pallasii* plants from South Kazakhstan and Kyrgyzstan. However, the plants from Altai and northern Kazakhstan differ morphologically from plants from southern Kazakhstan and Kyrgyzstan, with smaller flowers and longer pedicels, giving the impression of fewer flowers per inflorescence [21,22,23]. The filaments are also no longer than tepals, in contrast to plants from southern Kazakhstan and Kyrgyzstan, which have long filaments. Despite such obvious morphological differences, many botanists regard *A. pallasii* as a highly variable species [21,22], and some [23], having discovered significant karyological and morphological differences between plants from northern Kazakhstan and Kyrgyzstan, have not dared to recognize them as distinct species. *Allium pallasii* is throughout the distribution area diploid, with 2 n = 16 chromosomes [23,24,25].

Preliminary ITS sequences of plants from the Kurai steppe (Altai, Russia) and Kyrgyzstan displayed surprisingly different sequences. First investigations into taxonomy showed the great complexity of the taxonomy and nomenclature of *A. pallasii* as shown by the seven synonyms (*A*. *tenue* G.Don, *A*. *lepidum* Ledeb., *A*. *caricifolium* Kar.et Kir.,, *A*. *nitidulum* Fisch. ex Ledeb., *A*. *alberti* Regel, *A semiretschenskianum* Regel, and *A*. *saxatile* Hohen ex Boiss) in *Plants of the World Online* [26]. 

To shed light on these phylogenetic and taxonomic problems, plants from the entire distribution area of *A. pallasii* s. l. were systematically collected from 2010 and included in the geographical, morphological, cytological, and molecular analysis. 

## 2. Results

### 2.1. Morphology and Distribution 

**Figure 1:**
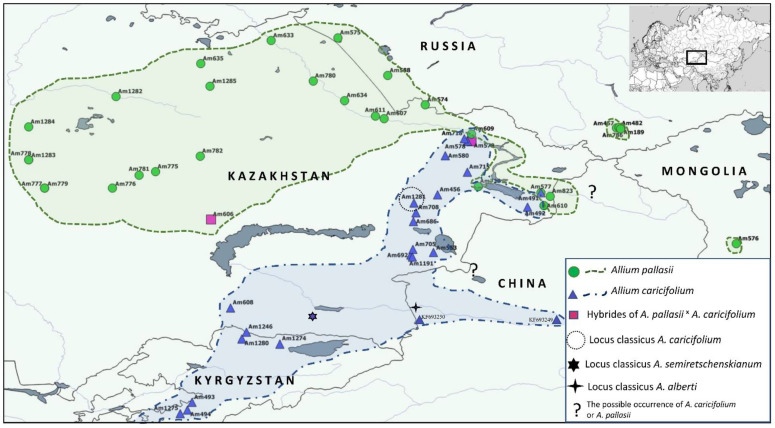
The geographic location of collected accessions and distribution of *A. pallasii* and *A. caricifolium*.

**Figure 2:**
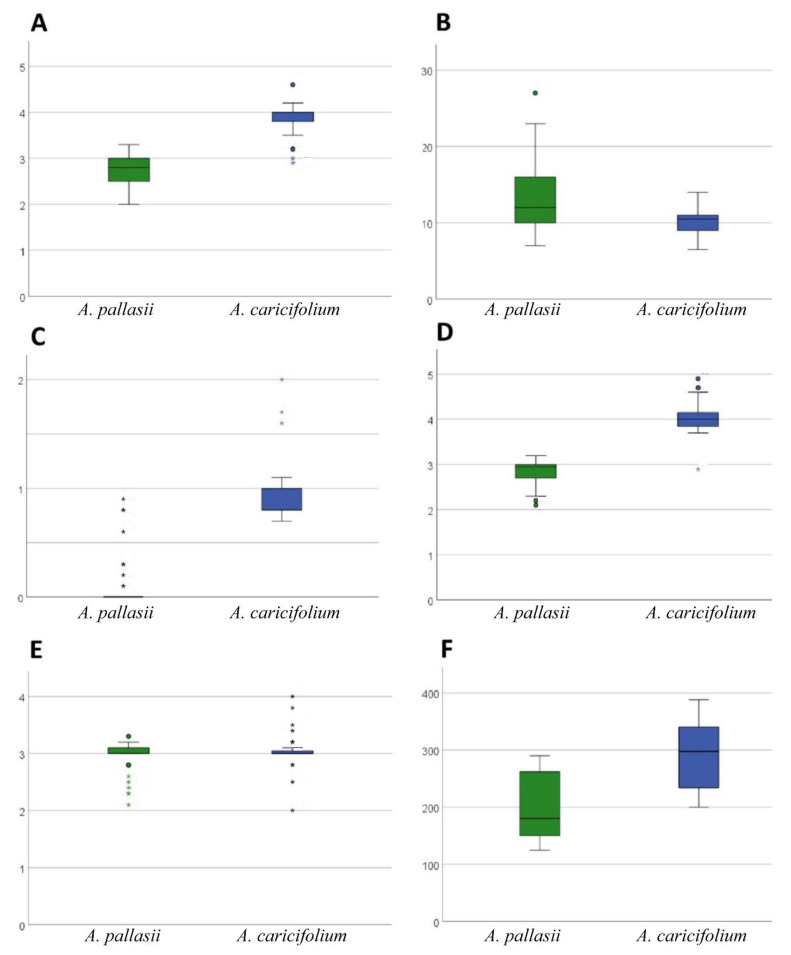
Boxplot analysis of different morphological character (in mm). (**A**): Carpel length, (**B**): Pedicel length, (**C**): Length of filaments outside tepals, (**D**): Filaments length in total, (**E**): Tepal length, (**F**): Scape length. The sizes range between 0–30 mm (**A**–**E**), the scape length is between 0 and 400 mm (**F**). *A. pallasii* is shown in green, *A. caricifolium* in blue.

**Figure 3:**
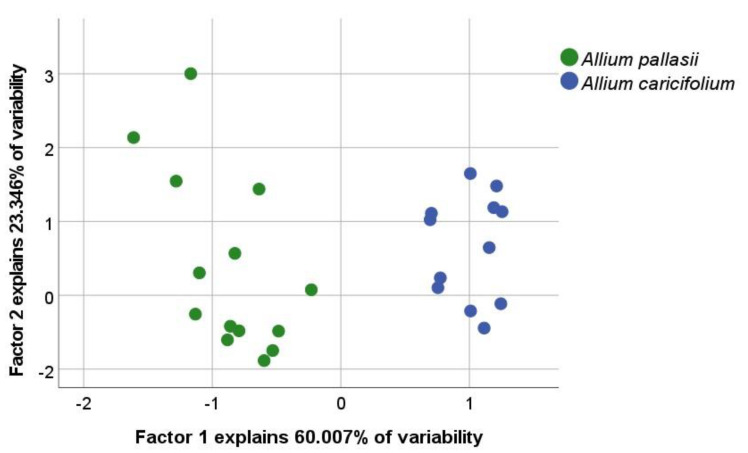
PCA analysis of morphological characters, shown in Table 1.

**Figure 4:**
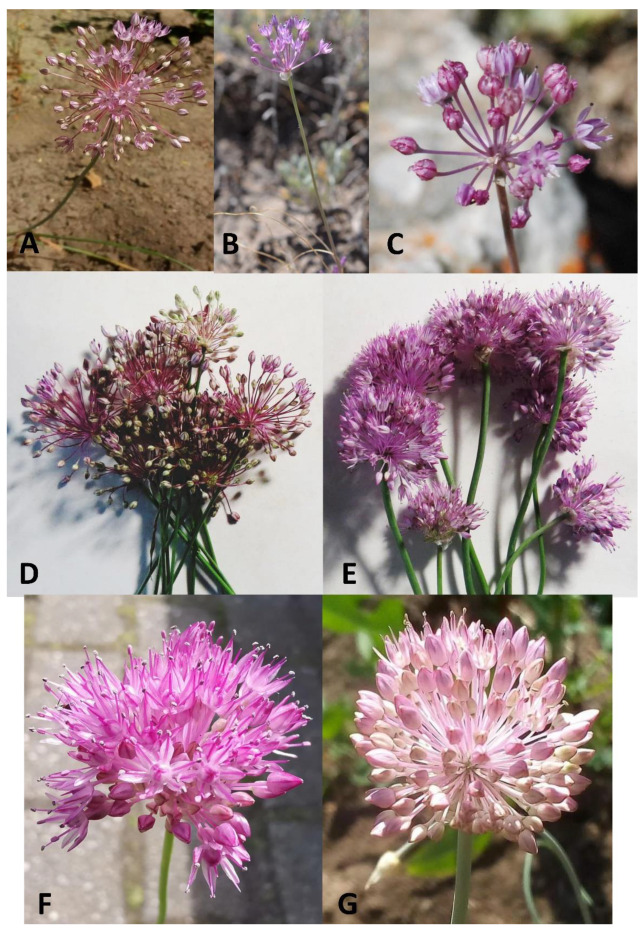
Inflorescences of the *Allium pallasii*: (**A**)—Am588 (Photo S. Smirnov); (**B**)—Am780; (**C**)–Am189; (**D**). Am607; *Allium caricifolium*: (**E**)—*Am708*; (**F**)—Am580; (**G**)—Am494. (All photos, except Am588, by N. Friesen, origin of the accessions in Appendix A). Accessions Am607 (**D**) and Am708 (**E**) were used to determine relative DNA amount.

Morphological analysis of herbarium sheets and our collections of the *A. pallasii* accession revealed a clear dividing line between the two morphotypes in the *A. pallasii* s.l.: plain steppe morphotypes and mountain steppe morphotypes. To the north of Lake Balkhash and to the east of Lake Zaysan *Allium pallasii* s. str. is widespread in plain steppe and south of this line only the mountain morphotype (*A. caricifolium*) with taller stems, dense inflorescence, and filaments 1.25 times longer than tepals, is found (Figure 1). Both species have apparent morphological differences; a comparison with some morphological features is presented in Table 1 and can be seen in Figure 2, Figure 3 and Figure 4.

### 2.2. Phylogenetic Analyses

#### 2.2.1. Position of *A. pallasii* s.l. in the Third Evolution Line

**Figure 5:**
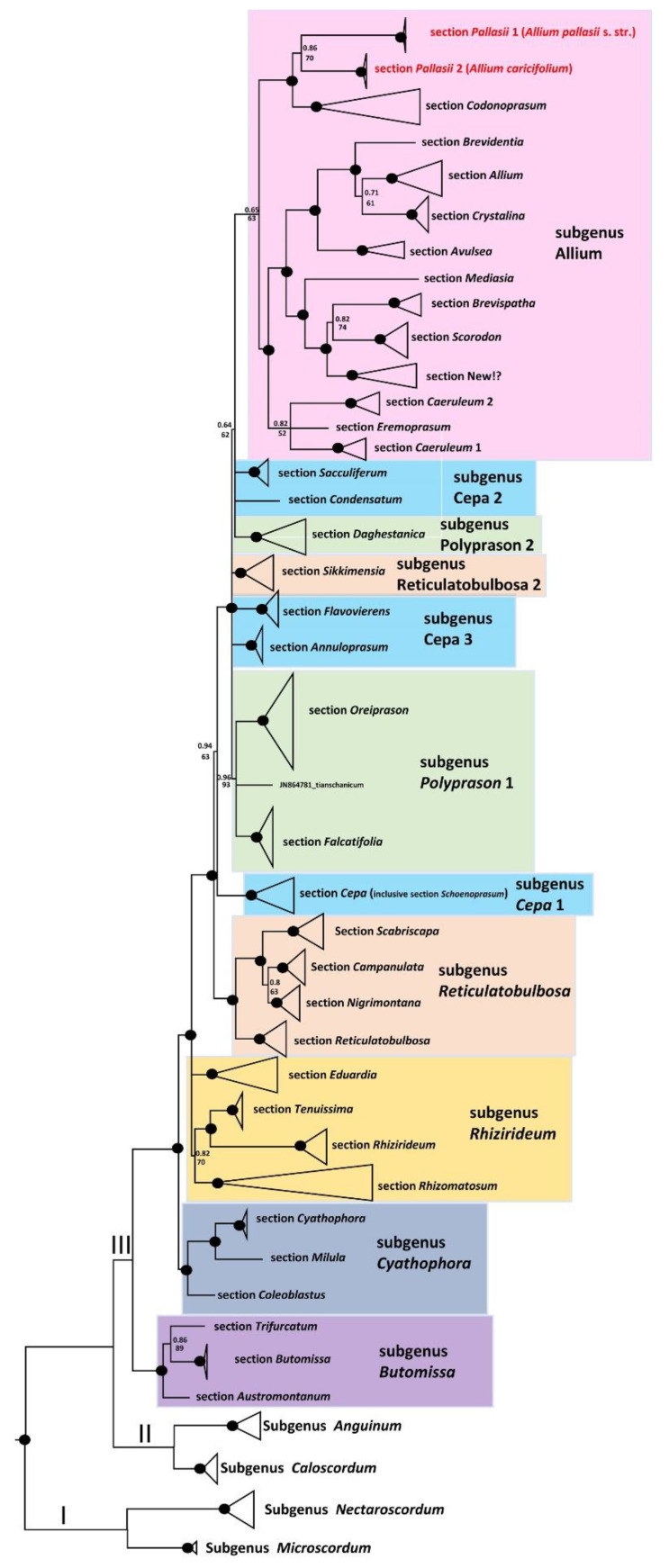
Generalized nrITS tree of the third evolutionary line of genus *Allium*. Numbers by nodes represent bootstrap support (1000 replicates) and Bayesian probabilities. Roman numerals (I, II, and III) designate clades of three evolutionary lines. The joint presence of Bayesian probabilities over 0.98 and bootstrap support over 95% is indicated with a black dot. *Allium pallasii* and *A. caricifolium* clades are sister. Marked red in the tree. For the origin of samples without GenBank accession numbers, see Appendix A.

The alignments of nrITS sequences (including the 5.8S gene) with 253 accessions of *Allium* species, a selection of representatives from each subgenus, and sections of the third evolutionary line, including five accessions of *A. pallasii* s. str. and five accessions from South Kazakhstan and Kyrgyzstan (*A. caricifolium*), consist of 732 characters of which 517 variable characters are parsimony informative. Unweighted parsimony analysis of the 253 sequences resulted in about 17 million most parsimonious trees of 4361 steps (CI = 0.2740). The substitution model TVM + G was chosen by AIC in JModeltest-2.1.7 for the Bayesian analysis. The Parsimony and Bayesian analyses produced identical topology (See Figure S2). All *A. pallasii* s. l. accessions are divided into two sister groups and stand surprisingly as a sister group to the *A.* sect. *Codonoprasum* of subgenus *Allium.* Both species (*A. pallasii* and *A. caricifolium*) are sister groups also in the plastid tree (*rpl*32-*trn*L) with 115 accessions from most sections of the third evolutionary line and five accessions from *A. pallasii* s.l. Both clades of *A. pallasii* and *A. caricifolium* together are entitled as a sister clade to *A.* sect. *Codonoprasum* (See Figure S3). The generalized ITS tree with sections and subgenera names is shown in Figure 5. Some subgenera in the third evolution line after classification [7] are not monophyletic; this applies to subgenera *Cepa*, *Reticulatobulbosa*, *Polyprason, Rhizirideum,* and possible *Allium*. These results agree with previously published phylogenetic analyses [5,9,11,12,13,27,28]. The phylogenetic consequences for the non-monophyletic subgenera should be made in the future with detailed analysis, but here is the most important finding for us, that the *A.* sect. *Pallasii* is a sister group to *A.* sect. *Codonoprasum* with strong support. The matching of the two sister clades *A. pallasii* and *A. caricifolium* is only moderately supported: Bayesian posterior probabilities (PP) = 0.86 and bootstrap support (BS) = 70.

#### 2.2.2. Phylogeny of *Allium pallasii* s.l.

Furthermore, we made a phylogenetic screening with 51 accessions *A. pallasii* s.l., carried out from the entire distribution area (31 accessions of *A. pallasii* and 20 accessions of *A. caricifolium*) with three representatives of the *A.* sect. *Codonoprasum* (*A. flavum* L., *A. paniculatum* L. [29,30] and *A. praescissum* Rchb. [31]) as outgroup with nuclear (ITS) fragments and two plastids (*trn*L-*rpl*32 and *trn*Q-*rps*16). ITS sequences within *A. pallasii* and *A. caricifolium* are monomorphic, with rare single-nucleotide swaps. Especially the accessions of *A. pallasii* s. str. have identical sequences. Only in the mountainous morphotype (*A. caricifolium*), are the accessions from the Alai Mountains in Kyrgyzstan grouped into a clade with relatively good support. Both species are divided into two sister groups with very high support because the sequences are very different (See Figure S3a). In the BLAST analysis, the nrITS sequences from *A. pallasii* s. str. were only 84.04% similar to *A. caricifolium* sequences from Northwest China (as *A. pallasii* in NCBI GenBank: GQ181077 China; KF693249 China: Xinjiang, Urumchi; KF693250 China, Xinjiang, Zhaosu), which correlates well with the group mean distance between *A. pallasii* and *A. caricifolium* ITS sequences (P = 0.188). 

**Figure 6:**
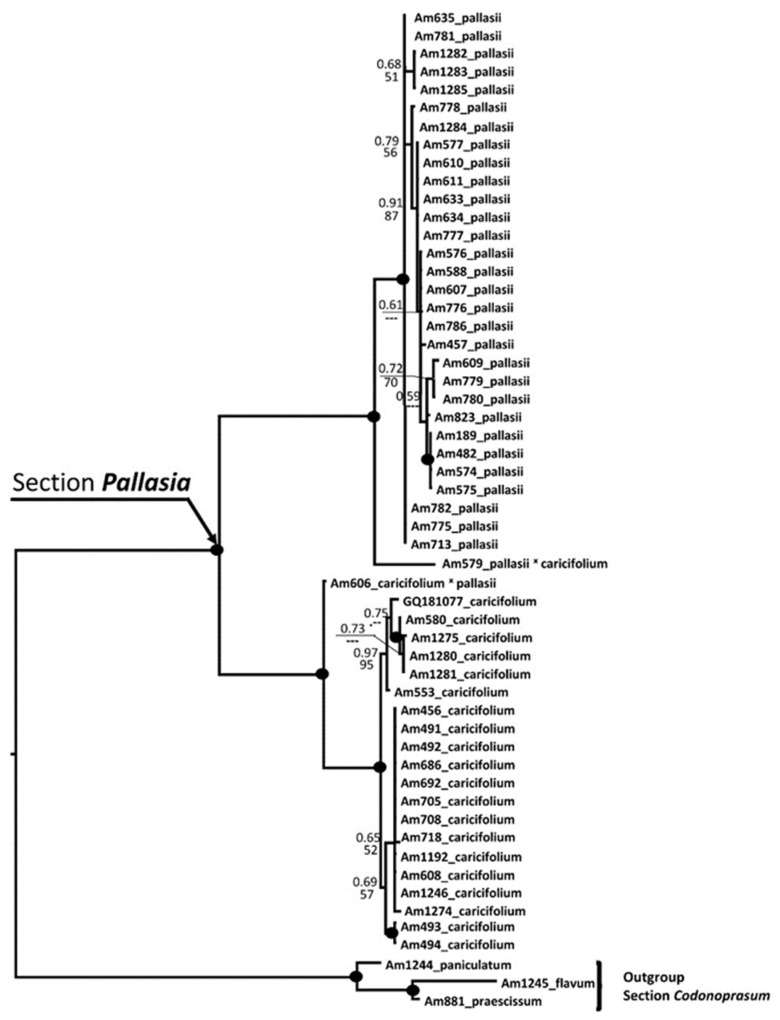
Combined nrITS and plastid (*trn*L-*rpl*32 and *trn*Q-*rps*16) tree of *A.* sect. *Pallasia*. Species from *A.* sect. *Codonoprasum* has been used as an outgroup. Numbers by nodes represent bootstrap support (1000 replicates) and Bayesian probabilities. The joint presence of Bayesian probabilities over 0.98 and bootstrap support over 95% is indicated with a black dot. The origin of samples is seen in Appendix A.

We obtained similar results with plastid sequences, where the polymorphism within morphotypes is significantly higher than with nrITS sequences. See the plastid tree in Figure S3b. There are only two discrepancies regarding the position of accession Am579 and Am606. In the ITS tree accession Am579 stays within *A. pallasii* s. str. clade and in the plastid tree clearly below the *A. caricifolium* clade. The situation at accession Am606 is reversed (Figure S3). This is a clear indication of the hybrid origin of these accessions. In addition, their location in the border regions between both species supports the hybridogenic origin (Figure 1). Except for these two cases, the topology of the trees is very similar, so we aligned and analysed all the sequences together (Figure 6). Both hybrid accessions are expected to stand apart in the tree, but both sister clades are clearly monophyletic with very strong support. There are a few small groupings in *A. pallasii* clade with weak support; only one subclade with four accessions (Am189, Am482, Am574, Am575) has strong support (PP = 0.97; BS = 95). All these accessions are from the easternmost distribution. In the BLAST analysis, the *trn*L-*rpl*32 spacer sequences from *A. pallasii* s. str. were only 94.19% similar to *A. caricifolium* sequence from Northwest China (as *A. pallasii* in NCBI GenBank: MN648632 complete chloroplast genome).

Within *A. caricifolium* clade are two subclades with good support: accessions Am580, Am1275, Am1280 and 1281 form one, and two accessions from Alai valley in Kyrgyzstan are the second well-supported clade. In the first subclade, two accessions are from Chu valley in Kyrgyzstan, one from Alay valley (Am1275), and one (Am580) is from the western part of the Zaysan lowland.

### 2.3. Cytology, Flowcytometry

**Figure 7:**
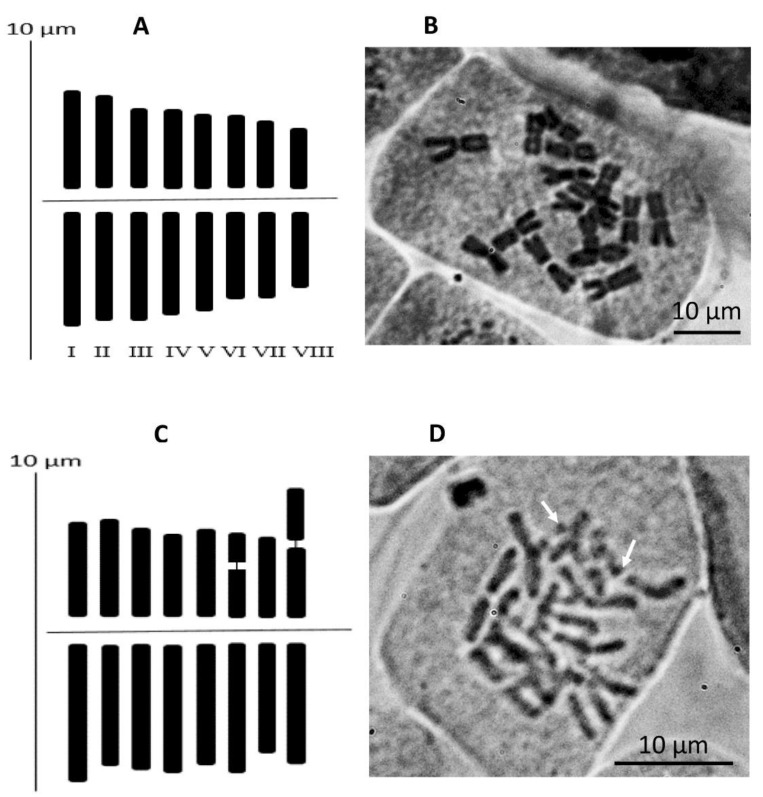
(**A**)—Idiogram of *Allium pallasii,* based on 36 metaphase plates; (**B**)—metaphase plate of Am804, (**C**)—Idiogram of *A. caricifolium* based on five metaphase plates; (**D**)–metaphase plate of Am1246. Arrows point to satellites. Roman numbers show the numbering of chromosome pairs.

From *A. pallasii,* we examined the karyotypes of four accessions (Am457, Am588, Am776, Am781). All four accessions have similar chromosome morphology. Two middle pairs of chromosomes have very small dot satellites in the shorter arm. There are only metacentric chromosomes in the karyotype of *A. pallasii*. Therefore, we calculated a combined idiogram of 36 metaphases (Figure 7a, Table 2. For *A. caricifolium,* we could study the chromosome morphology of the accession Am1246. The sixth pair of chromosomes are metacentric, and two satellite chromosomes are submetacentric. Compared to *A. pallasii*, the satellites are massive in *A. caricifolium*, between one and two µm (Figure 7b, Table 3). Overall, the chromosomes in *A. caricifolium* are also slightly larger. Total karyotype diploid length (TKL) in *A. pallasii* = 87.14 µm and in *A. caricifolium* = 103.19 µm. This correlates well with the estimated genome size by flow cytometry in both species: *A. pallasii* 2C = 14.03 pg (Am607); *A. caricifolium* 2C = 20.37 pg (Am708). See the histograms of relative DNA content in Figure S5.

### 2.4. Nomenclatural Remarks

Murray [17] described *A. pallasii* on the plants grown in the botanical garden of the University of Göttingen from seeds, sent by P.S. Pallas, without geographical origin. According to the description and analysis of the picture [17] (Table 1) and type material, the name *A. pallasii* belongs to the plain steppe morphotypes of plants. The species named *A. tenue* G.Don [34] is also based on the samples from the Herbarium of Pallas. The description staminibus perianthio aequalibus” clearly refers to it as a synonym of *A. pallasii* s. str. The species named *A. lepidum* for the plain steppe morphotypes was donated by Ledebour [35] that included in the protologue a short description and an illustration (Table CCCLV) strangely named *A. pallasii*. The other synonym name for plain steppe morphotypes in POWO [26] is *A. nitidulum* Ledeb. 

This name is a nomen nudum cited by Ledebour [36] as “*A. nitidulum* Fisch. in herb. reg. berol.” as synonym of *A. pallasii*. That means Ledebour, in both cases (*A. lepidum* and *A. nitidulum*), did not recognize the species status for Altai plants, but ultimately included them in synonyms to *A. pallasii*. Only Regel [37] first validated the name “*A. nitidulum*” as a variety of *A. pallasii*. The name of *A. saxatile* Hohen. ex Boiss. (nom.illeg.) was also incorrectly cited as synonym to *A. pallasii* [26]. It is a synonym of *A. kunthianum* Vved. [21].

All other names regarded as synonyms of *A. pallasii* s. l. (i.e., *A. caricifolium*, *A. alberti*, and *A. semiretschenskianum*) belong to the southern mountain morphotype with priority name *A. caricifolium*. *Allium caricifolium* Kar. & Kir. is described on the plants collected in the Mountains near the Ajagus settlement. The morphological character in the description “… staminibus simplicibus, basi subuîatis, perigonium subdupio excedentibus“ unequivocally refers to the mountainous morphotype from south-east Kazakhstan and Kyrgyzstan [38], and is typified by [39] (lectotype MW0591659). Regel had correctly identified the differences between *A. pallasii* plants and the plants from southern Kazakhstan and described the plants from the Almaty region (formerly Vernoe) as *A. semiretschenskianum* [40]. The name *A. caricifolium* Regel had been unfortunately placed as a synonym for *A. pallasii* [41]. The situation with the name *A. alberti* Regel [41] is a bit complicated. This species was described from plants grown in the garden from bulbs collected by Albert Regel in the Chinese part of the Ili River in 1876 [42]. The lectotype of *A. alberti* (LE01010227, designated by [43]) shows a bulbless plant, and the morphological characters show extreme similarity with *A. caricifolium* and *A. semiretschenskianum* (Lectotype of *A. semiretschenskoanum* LE000518202 designated here, LE00052546). But Regel’s original description of *A. alberti* is slightly confusing. He gives a detailed description of the slender reticulate-fibrous outer tunics of the bulbs “Bulbi ovati tunicis exterioribus tenuibus totis reticulate-fibrosis, …” and gives *A. moschatum* L. and *A. sindjarense* Boiss. & Hausskn. ex Regel (*A.* sect. *Scorodon*) as related species. All other characteristics in the description match *A. caricifolium* very well. We can only guess whether, or not, this is a mix-up with another bulb that his son Albert Regel [42] collected during his trip to China. It was Vvedensky [21] who put both species *A. semiretschenskianum* and *A. alberti* as synonyms to *A. pallasii*, and we put these two names as synonyms to *A. caricifolium.*

Section *Pallasia* (Tzag.) F.O.Khass., R.M.Fritsch & N.Friesen—2017: 87 [44]. Khasanov F. in Sennikov (ed.) Flora of Uzbekistan. Vol 1. ≡ Section *Pallasia* (Tzag.) F.O.Khass., R.M.Fritsch & N.Friesen in [7], nom. invalid. ≡ *Allium* ser. *Pallasia* Tzag. Bot. Mater. Gerb. Bot. Inst. Bot. Acad. Nauk Kazakh. S.S.R. 11: 44 (1979 [45]).

Type: Allium pallasii Murray

*Allium pallasii* Murrey, Novi Comment. Soc. Regiae Sci. Gott. vi. (1775) 32. t. 3.—Lectotype (designated by 45: 87): Herb. Murrey. Described from plants grown in the botanical garden of the University of Göttingen from seeds, sent by P.S. Pallas. without geographical origin. (MW barcode MW0591688; image of the lectotype available at https://plant.depo.msu.ru/open/public/item/MW0591688, accessed on 1 April 2022) 

= *A. lepidum* Ledeb. 1833. Icon. Pl. 4: 17, Table CCCLV.

Type ? Ilab. in collibus apricis et campestribus siccis ad fl. Irtysch a fortalitio Ustkamenogorsk usque ad lacum, qui Noor—Saisan vacatur. Fl. 3 Iajo. 7 J.

=*A. tenue* G.Don, Mem. Wern. Nat. Hist. Soc. vi. (1827): 34.

Type ? The description of this plant, and the preceding one, were taken from specimens in the Herbarium of Prof. Pallas, now in the possession of Mr. Lambert [34].

=*A. pallasii* var. *nitidulum* (Fisch.) Regel, Trudy Imp. S.-Petersburgs. Bot. Sada 10: 317 (1887).

Type: Herb. Fischer. No. 127. Collected near the Chuya River in meadows. July of the 10th day (LE!)

*Description*—Bulb ovoid, 12 mm diam., with outer gray, almost leathery shells. Shells with clear parallel veins. Stems 120–200 (290) mm high, covered by leaf sheaths up to 1/3–1/2 of its length. Leaves 2–3, filiform, semi-cylindrical, smooth, shorter than stem. Spathe 2 (3) times shorter than umbel, shortly pointed. Inflorescence is hemispherical or more often spherical, many-flowered, loose. Pedicels are almost equal between themselves, 2–3 (4) times longer than perianth. The tepals are pink, with a purple vein, shiny, 3 mm long, equal in length, lanceolate, and acute. The filaments of the stamens do not exceed the length of the perianth, subulate, and are slightly widened internally at the base. The style of the pistil is equal to or slightly longer than the perianth.

*Distribution*—Central and Northeast Kazakhstan, West Siberia (Kulunda Steppe), and West Altai. Two isolated distributions are in the Kurai Steppe (southeast Altai Mountains) and in Dzungarian Gobi (West Mongolia).

*Habitat*—In sandy and fescue steppes, on salt licks

*Allium caricifolium* Kar. et Kir., Bull. Soc. Imp. Naturalistes Moscou 14: 854 (1841).

Lectotype (designated by 39: 39). In montosis sterilibus prope Ajagus, nec non in apricis montium Aktschauly et Tarbagatei ad torrentes Dschanybek et Terekty. Leg. Karelin et Kirilov a. 1840. (MW! Barcode MW0591659!, the image of the lectotype available at https://plant.depo.msu.ru/open/public/item/MW0591659, accessed on 1 April 2022)

=*A. semiretschenkianum* Regel, Trudy Imp. S.-Peterburgsk. Bot. Sada v. (1877) 630.

Lectotype: Balchasch s.d., s. coll. (LE! Barcode LE000518202).

=*A. albertii* Regel 1878 in Acta Horti Petrop. 5: 632.

Type: Bulbe leg. A. Regel prope Suidun ad fluvium Ili. Ex horto bot. Petropolitani. 78.5. (LE! LE01010227)

*Description*—Bulb ovoid, 10–20 mm thick, outer shells gray, papery, without veins. Stem 20–65 cm high, 1/3 or almost up to 1/2 covered with smooth leaf sheaths. Leaves 3–4, filiform or narrowly linear, 1.5 (2.5) mm wide, shorter than the stem. Spathe 2–3 times shorter than an inflorescence, shortly pointed. Inflorescence spherical, many-flowered, dense. Pedicels are almost equal, 2–3 times longer than perianth. Tepals are pink with a purple vein, shiny, 3–4 mm long, equal, lanceolate or oblong-lanceolate, and acuminate. Filaments of stamens up to 1.5 times as long as tepals, subulate from a triangular base, inner base wider than the outer ones. The style of the pistil is slightly longer than the perianth.

*Distribution*–To the west of the Zaisas basin, Tarbagatai, Dzungarian Alatau and Central and Eastern Tian Shan

*Habitat*–On fine earth, gravelly and rocky slopes, outcrops of variegated rocks in the mountainous and subalpine belt

## 3. Discussion

All of our results (morphological, geographical, cytological, and molecular) quite clearly confirm the presence of two very well separated species in the formerly monotypic *A.* sect. *Pallasia*: *A. pallasii* s. str., and *A. caricifolium*. Li et al. [9] erroneously introduced several other *Allium* species into the section, mostly belonging to *A.* sect. *Caerulea* [46]. Complete chloroplast genome analysis of seven Chinese species (*A. delicatulum, A. schoenoprasoides, A. songpanicum, A. tanguticum, A. caeruleum* and *A. teretifolium*, including *A. pallasii* (*A. caricifolium*) from northwest China [27]), supports the isolated position of section *Pallasia*. The plastid genome of Chinese *A. pallasii* (MN648632) and nrITS sequences (GQ181077, KF693249, KF693250) belong to the *A. caricifolium*. It is possible that *A. pallasii* s. str. also occurs in the border region east of the Black Irtysh River (see Figure 1). So far, we have seen no evidence of this.

We confirmed 2 n = 16 for both morphotypes as expected from earlier studies [23] for both species and for *A. caricifolium* [23,24]. Vakhtina & Kudryashova [23] studied the morphology of the chromosomes of both morphotypes (*A. pallasii* from North East Kazakhstan and *A. caricifolium* from Transili Alatau) and found that both karyotypes differ in the position and the size of the satellites in the satellite chromosomes. Our data confirm these differences. Differences in plant morphology were also well recognized, but unfortunately, no consequent conclusions were made [23]. Simply *A. pallasii* was declared as very polymorphic.

It is also very surprising that the sequences of both species are so different (only 84% similarities in ITS sequences) and still grouped as a sister subclade. The closest relationship to *A.* sect. *Codonoprasum* cannot be explained morphologically either. Morphologically *A. pallasii* and *A. caricifolium* are more like species from the *A.* sect. *Caerulea* (e.g., with *A. delicatulum, A. caesium,* and others), which explains the inclusion of some species by Li et al. [9] in *A.* sect. *Pallasia*. In the nrITS and plastid trees, the species from *A.* sect. *Caerulea* are relatively distant from *A.* sect. *Pallasia* (Figure 1, Figures S1 and S2). When comparing the genetic differences between *A. pallasii* and *A. caricifolium* with other *Allium* species where times of evolutionary splits were estimated [16,28], we hypothesize an Oligocene split between *A.* sect. *Codonoprasum* and *A.* sect. *Pallasii,* and between *A. pallasii* and *A. caricifolium* Myocene split. These splits can be explained by the vegetation/landscape history of the Zaysan Depression.

The Oligocene in extratropical Eurasia is marked by the expansion of the Boreal vegetation zone (warm and humid) and the formation of temperate deciduous mesophyllous coniferous-broadleaved forests (Turgai Flora) [47,48]. In East Kazakhstan, the Turgai Flora became dominant during the Oligocene and the first half of the middle Miocene [49,50]. During the Miocene, large depressions in the hilly zone of the present-day Altai and northern Tien Shan were formed, and an inland lake has been proved for the Zaysan Depression [49]. It is suggested that a paleolake existed here since the Cretaceous period and that the Zaysan Basin was never dried [51]. 

Present-day Altai and northern Tien Shan mountains are believed to be of relatively recent origin (Neogene) and started to develop from the Miocene onwards as a direct result of the far-field effects of the Himalayan collision [52]. With the rising mountains, the relief energy increased and had consequences for the drainage pattern. It is hypothesized that the Altai-draining rivers flew southwards into the Zaysan and adjacent Junggar Basin, and the Tien Shan-draining rivers northwards also into the Junggar Basin [53] filling the Zaysan paleolake and creating paleolakes in the Junggar Basin. The filling of the paleolakes culminated in a united Zaysan-Junggar Basin Paleolake, which in the Late Pliocene-Pleistocene cut through the northern end of the Zaysan Basin triggering the birth and the formation of the course of the Irtysh River [53]. 

This scenario has consequences for the vegetation history in the Zaysan Depression. Forest vegetation (Turgai Flora) and paleolakes prevented the establishment of modern steppes for a long time, and it would appear that the steppe occurred only recently. Unfortunately, there are no Pliocene and younger paleo records from the Zaysan Basin itself but several studies from neighboring regions such as the area near Semei on the Irtysh River and the Kulunda Steppe point to a late Pleistocene/early Holocene steppe vegetation [54,55]. 

Based on the climate/landscape history outlined above, we suggest the following scenario of the evolutionary history of our vicarious species *Allium pallasii* and *A. caricifolium*: The original distribution area of the ancestral species was separated with the emergence of the Altai orogeny into two disjunct areas, leading to allopatric speciation. *Allium pallasii* s.str. survived in the Altai mountains (Kurai Steppe) and *A. caricifolium* in the Tian Shan and Tarbagatai mountains. With floods after the breaching of the dams of Chuya and Kurai lakes in Altai after the Ice Age [56,57], the seeds of *A. pallasii* were spread to the Kulunda Steppe and from there dispersed very quickly in the steppe of northern Kazakhstan. This could explain why the ITS sequences of all accessions of *A. pallasii* are identical. *Allium caricifolium* may have persisted in several places in the Tian Shan Mountains and spread north and east after the Ice Age, where it met with *A. pallasii* at Lake Zaysan. Similar splits between northern Kazakhstan, western Siberia, including the right bank of the Irtysh River up to the Altai mountains in one site, and mountainous regions in south-eastern Kazakhstan, west of Zaysan Lake in the second, have recently been discovered and molecularly confirmed in other taxa of the genus *Allium*: sect. *Oreiprason* [58]; *Allium tulipifolium* Ledeb. and *A. robustum* Kar. et Kir. [28]; *A. obliquum* L. [59], and also in other plant groups: genera *Krascheninnikovia* (family Amaranthaceae) [60] and *Goniolimon* (family Plantaginaceae) [61]. All this confirms the complex phylogenetic history of the steppe flora [62,63].

## 4. Materials and Methods

### 4.1. Morphological and Distribution Analyses

We compiled distribution maps from literature and online databases and analysed herbarium collections, including field collections. A total of 20 individuals of *A. caricifolium* from 4 Herbarium sheets were analyzed for the morphological analysis [Am705, Am715, Am1192 (see the origin in Appendix A), Am1281 OSBU-24372 (47°33′27″ N, 80°37′21″ E)]. A total of 22 individuals of *A. pallasii* were analyzed from 5 Herbarium sheets [Am482, Am780, Am1285 (see the origin in Appendix A), and OSBU 24849 (48°12′58″ N, 69°13′16″ E), OSBU 25938, (51°27′50″ N, 74°19′7″ E) The average with the associated standard deviation was calculated from the measurements. The measurements on the plants were made using a ruler and a magnifying glass. The data were analysed in the SPSS program (Version 28 https://www.ibm.com/products/spss-statistics accessed on 22 April 2022). A boxplot and PCA analysis were executed with this program. The PCA is based on a correlation matrix of characters (Table 1) using the Pearson correlation coefficient. In addition, a Kolmogorov–Smirnov test for normal distribution was carried out beforehand.

Published data were critically evaluated by reference to herbarium material deposited in ALTB, AA, BRNO, FRU, HAL, GAT, LE, M, MHA, TK, MW, NS, NSK, OSBU, TASH, XJA, and W [64,65]. Herbarium acronyms are according to the Index Herbariorum [66]. 

### 4.2. Taxon Sampling

Bulbs and leaf samples of more than 50 accessions of *A. pallasii* s. l. for DNA isolation were collected in the course of several collecting trips in Russia (Altai), Mongolia, and Kazakhstan from 2010 and growing in the Botanical Gardens in Osnabrück (Germany) and Barnaul (Russia). Some accessions of DNA were isolated from Herbarium sheets.

Newly sequenced accessions are marked with Am number in the trees, and their origin is shown in Appendix A. To determine the position of the *A. pallasii* in the genus, we took the available nuclear ITS sequences and *rpl*32-*trn*L (UAG) plastid fragment of accessions of the species with representatives from all sections of the third evolution line while some accessions from the first and second evolution lines were selected as the outgroup [7]. Sequences from NCBI GenBank (https://www.ncbi.nlm.nih.gov/nucleotide/ accessed on 3 December 2021) are marked with GenBank accession numbers on the trees. 

### 4.3. DNA Extraction, Amplification and Sequencing

Total genomic DNA was isolated from leaves in silica gel using the InnuPREPP Plant DNA Kit (Analytic Jena AG) according to the manufacturer’s instructions and used directly in PCR amplification. The complete nuclear ribosomal ITS region (ITS1, 5.8S and ITS2) was amplified using the primers ITS-A [67] and ITS-4 [68]. The PCR conditions for ITS followed ref. [7]. PCR conditions and primers for the chloroplast regions *trn*L-*rpl*32 and *trn*Q-*rps*16 were described in [69]. PCR products were sent to Microsynth SeqLab (Balgach, Switzerland for sequencing. The sequences from all the individuals were manually edited in Chromas Lite 2.1 (Technelysium Pty Ltd.South Brisbane, Australia) and aligned with ClustalX [70], the alignment was manually corrected using MEGA 7 [71]. 

### 4.4. Phylogenetic Analyses

Both data sets (nrITS and the cpDNA *trn*L-*rpl*32 markers) for identifying the position of *A.* sect *Pallasia* in the third evolution line and to find the closest relatives of *A. pallasii* were analysed separately through Fitch parsimony with the heuristic search option in PAUP version 4.0 b10 [72]) with MULTREES, TBR branch swapping and 100 replicates of random addition sequence. Gaps were treated as missing data. The consistency index (CI) [73] was calculated to estimate the amount of homoplasy in the character set. The most parsimonious trees returned by the analysis were summarized in one consensus tree using the strict consensus method. Bootstrap analyses (BS) using 1000 pseudoreplicates were performed to assess the support of the clades [74]. Bayesian phylogenetic analyses were also performed using MrBayes 3.1.23 [75]. The sequence evolution model was chosen following the Akaike Information Criterion (AIC) obtained from jModelTest2 [76]. Two independent analyses with four Markov chains were run for 10 million generations, sampling trees every 100 generations. The first 25% of trees were discarded as burn-in. The remaining 150,000 trees were combined into a single data set, and a majority-rule consensus tree was obtained along with posterior probabilities (PP). To determine molecular variability throughout the range, more than 50 accessions of *A. pallasii* s.l. and three species from the *A.* sect. *Codonoprasum* as outgroup, nrITS, and two noncoding regions plastid DNA (*trn*L-*rpl*32, *trn*Q-*rps*16) were sequenced and analysed as above. The group mean distance (P) was estimated with MEGA7.

### 4.5. Cytology, Flowcytometry

Bulbs were planted in pots, and growing roots were used for the karyotype analysis. Root tips were excised from the bulbs and kept overnight in distilled water on ice. They were then transferred to room temperature for 20 min and pre-treated for 3 h at room temperature in an aqueous solution of 0.1% colchicine. Roots were then fixed in a freshly prepared mixture of 96% ethanol and glacial acetic acid (3:1 *v*/*v*). Root tips were stained using hematoxylin according to the protocol reported by Smirnov [77]. Well-spread metaphase plates were electronically documented (digitally photographed), and finally, the chromosomes of the best plates were measured and pairwise arranged using the KaryoType software [78]. For *A. caricifolium*, 5 metaphase plates from one individual were evaluated (Am1246, Appendix A), for *A. pallasii*, 4 individuals were used (Am776, Am781, Am588, Am457, Appendix A), which provided 3–12 usable metaphase plates. The measurements from all metaphase plates were combined here, a total of 36 metaphase plates. Because the idiograms automatically assembled by the software were not satisfactory, we manually ordered the chromosome pairs according to their length and shape. The idiograms were designed using the bar graph function implemented in MS Excel^®^. The terminology of [32,33] was applied. 

Flow cytometry was used for the determination of DNA amount. Fresh leaf material was harvested, and ca. 0.5 cm^2^ leaf material was chopped with a sharp razor blade in a Tris MgCl_2_ buffer supplemented with propidium iodide (50 μg/mL), RNase (10 μg/mL), and 2-mercaptoethanol (0.2%) [79]. The samples were filtered through a 50-μm nylon membrane into a sample tube. Subsequent flow cytometry was performed on a Partec CyFlow PA (Partec, Münster, Germany). As an internal standard *Pisum sativum* ‘Ctirad’, 2 C = 9.09 pg was used [80]. Histograms were analysed using the Flowing Software 2.5.1. (Turku Bioscience Centre, Turku, Finland).

## Figures and Tables

**Table 1:** Comparison of morphological characters of *A. pallasii* (20 individuals) and *A. caricifolium* (22 individuals). Quantitative characters are expressed in mm and presented as mean ± standard deviation (extreme values in brackets).

Character	*A. pallasii*	*A. caricifolium*
Scape length	194.7 ± 55.3 (125–290)	275 ± 63 (200–388)
Pedicel length	12.9 ± 3.7 (7.5–19)	10.4 ± 1.6 (6.5–14)
Tepals length	2.9 ± 0.1 (2.1–3.3)	3 ± 0.2 (2.0–4.0)
The ratio of tepals length/pedicels length	1.1	1.2
Length of filaments outside the tepals	0.1 ± 0.1 (0)	1 ± 0.1 (0.7–1.1)
Filaments length in total	2.9 ± 0.1 (2.6–3)	4 ± 0.1 (3.8–4.3)
The ratio filaments length outside the tepals/total length	1.02	1.25
Carpel length	2.8 ± 0.1 (2–3.3)	3.9 ± 0.1 (2.9–4.6)
Flowers per inflorescence	36.8 ± 17.5 (22–75)	52.8 ± 12.9 (37–66)

**Table 2:** Karyo-morphometric parameters of A. pallasii accessions in µm.

Pair No.	TAL (µm)	RL %	LA (µm)	SA (µm)	Sat	CI %	Type
1	6.6 ± 0.8	15.1 ± 0.2	3.5 ± 0.4	3.0 ± 0.5	0	46.3 ± 1.4	m
2	6.2 ± 0.7	14.3 ± 0.2	3.4 ± 0.3	2.9 ± 0.4	0	46.1 ± 2.0	m
3	5.9 ± 0.7	13.6 ± 0.2	3.4 ± 0.4	2.6 ± 0.4	0	43.4 ± 1.1	m
4	5.6 ± 0.9	12.9 ± 0.4	3.2 ± 0.5	2.4 ± 0.4	0	43.2 ± 1.1	m
5	5.4 ± 0.7	12.3 ± 0.1	3.1 ± 0.3	2.3 ± 0.5	0	42.8 ± 2.6	m
6	5.0 ± 0.6	11.5 ± 0.3	2.8 ± 0.2	2.2 ± 0.4	0	44.5 ± 2.4	m
7	4.7 ± 0.6	10.8 ± 0.1	2.7 ± 0.4	2.1 ± 0.3	0	43.8 ± 1.5	m
8	4.2 ± 0.6	9.6 ± 0.1	2.3 ± 0.2	1.9 ± 0.4	0	44.4 ± 2.6	m

Abbreviations: TAL total absolute length; RL relative length; LA long arm; SA short arm; Sat satellite; CI centromeric index; Type chromosome nomenclature according to [32,33]; TKL total karyotype diploid length. MCA mean centromeric asymmetry; KCI Karyotype centromeric index. *A. pallasii*: TKL = 87.1 ± 11.6; MCA = 11.4 ± 2.5; CI_total_ = 44.3 ± 1.3.

**Table 3:** Karyo-morphometric parameters of A. caricifolium in µm.

Pair No.	TAL (µM)	RL %	LA (µM)	SA (µM)	Sat	CI %	Type
1	7.2 ± 1.4	13.9 ± 2.5	4.3 ± 0.7	2.9 ± 0.9	0	40.7 ± 8.7	m
2	6.7 ± 1.2	13.1 ± 2.1	3.7 ± 0.6	3.0 ± 1.1	0	44.6 ± 11.4	m
3	6.6 ± 1.1	12.9 ± 1.9	3.9 ± 0.7	2.8 ± 0.5	0	41.6 ± 4.0	m
4	6.5 ± 0.3	12.5 ± 1.1	3.9 ± 0.3	2.5 ± 0.3	0	39.1 ± 4.5	m
5	6.4 ± 0.5	12.4 ± 0.9	3.7 ± 0.3	2.7 ± 0.5	0	41.9 ± 5.9	m
6	6.4 ± 0.8	12.4 ± 1.2	3.7 ± 0.4	1.8 ± 0.8	1	27.4 ± 8.1	m
7	5.9 ± 1.3	11.4 ± 2.0	3.4 ± 0.7	2.5 ± 0.6	0	42.4 ± 5.6	m
8	5.9 ± 1.0	11.4 ± 2.5	3.7 ± 0.7	2.2 ± 0.6	2	36.7 ± 7.6	m

Abbreviations: TAL total absolute length; RL relative length; LA long arm; SA short arm; Sat satellite; CI centromeric index; Type chromosome nomenclature according to [32,33]; TKL total karyotype diploid length. MCA mean centromeric asymmetry; KCI Karyotype centromeric index. *A. caricifolium*: TKL = 103.2 ± 5; MCA = 18.9 ± 4.8; CI_total_ = 40.5 ± 5.3.

## Data Availability

Relevant data applicable to this research are within the paper.

## Supplementary Materials

The following supporting information can be downloaded at: https://www.mdpi.com/article/10.3390/plants11111465/s1, Figure S1. Phylogenetic tree of third evolutionary lineage of the genus Allium, based on ITS sequences from NCBI GenBank. Figure S2. Phylogenetic tree of third evolutionary lineage of the genus Allium, based on CP DNA sequences (trnL-rpl32) from NCBI GenBank. Figure S3. A—Phylogenetic tree of A. sect. Pallasia accessions, based on ITS sequences; B—Phylogenetic tree of A. sect. Pallasia accessions, based on two combined fragments of plastid DNA (trnL-rpl32, trnQ-rps16). Figure S4. Histograms of relative DNA content were obtained after analysis of nuclei isolated from young leaf tissues of A. pallasii, accession Am607 (A) and A. caricifolium, accession Am708 (B).

## Appendix A

**Table A1:** Origin, source, and GenBank accession numbers of *Allium* sequences used for phylogenetic analyses.

Accession	Art Name	Coordinates	Country	Voucher	rITS	trnQ-rps16	rpl32trnL
Am189	*Allium pallasii*	50°14′15″ N 87°57′34″ E	RU	OSBU: 18243	OM891893		
Am457	*Allium pallasii*	50°14′14″ N 87°48′40″ E	RU	OSBU: 22275	OM891894	OM983335	OM983382
Am482	*Allium pallasii*	50°14′38″ N 87°53′48″ E	RU	OSBU: 23174	OM891895		
Am574	*Allium pallasii*	51°2′49″ N 81°2′16″ E	KZ	NS: 0012843	OM891896		
Am575	*Allium pallasii*	53°25′0″ N 77°57′0″ E	RU	NS: 0012841	OM891897	OM983336	OM983383
Am576	*Allium pallasii*	45°58′30″ N 92°14′59″ E	MN	ALTB: Smirnov, 19 June 1999	OM891898	OM983337	OM983384
Am577	*Allium pallasii*	47°55′60″ N 85°7′60″ E	KZ	ALTB: Smirnov, Antonjuk, 29 May 2000	OM891899	OM983338	OM983385
Am579	*Allium pallasii x A. caricifolium*	49°45′59″ N 82°38′9″ E	KZ	ALTB: Smirnov, Antonyuk, 20 May 2000	OM891900	OM983339	OM983386
Am588	*Allium pallasii*	52°4′48″ N 79°42′35″ E	RU	ALTB: Smirnov, 22 July 13	OM891901	OM983340	OM983387
Am607	*Allium pallasii*	50°33′40″ N 79°35′14″ E	KZ	OSBU: 23346	OM891902	OM983341	OM983388
Am609	*Allium pallasii*	49°54′58″ N 82°40′21″ E	KZ	TK: Prokopjev, 13 June 1982	OM891903	OM983342	OM983389
Am610	*Allium pallasii*	47°28′57″ N 85°14′25″ E	KZ	TK: Schischkin, 3 June 1914	OM891904		
Am611	*Allium pallasii*	50°38′47″ N 79°16′5″ E	KZ	TK: Prokopjev et al., 1 June 1975	OM891905		
Am633	*Allium pallasii*	53°19′0″ N 75°34′60″ E	KZ	NS: 0015875	OM891906	OM983343	OM983390
Am634	*Allium pallasii*	51°12′0″ N 78°10′60″ E	KZ	NS: 0015873	OM891907	OM983344	OM983391
Am635	*Allium pallasii*	52°29′55″ N 73°5′38″ E	KZ	NS: 0015872	OM891908	OM983345	OM983392
Am713	*Allium pallasii*	48°9′52″ N 82°54′23″ E	KZ	OSBU: 24384	OM891909	OM983346	OM983393
Am775	*Allium pallasii*	48°40′45″ N 71°29′20″ E	KZ	OSBU: 24800	OM891910	OM983348	OM983394
Am776	*Allium pallasii*	48°5′47″ N 69°57′58″ E	KZ	OSBU: 24841	OM891911	OM983349	OM983395
Am777	*Allium pallasii*	48°6′4″ N 67°34′3″ E	KZ	OSBU: 24890	OM891912	OM983347	OM983396
Am778	*Allium pallasii*	49°6′41″ N 66°59′48″ E	KZ	OSBU: 24875	OM891913	OM983350	OM983397
Am779	*Allium pallasii*	48°6′4″ N 67°34′3″ E	KZ	OSBU: 24898	OM891914	OM983351	OM983398
Am780	*Allium pallasii*	51°53′29″ N 77°3′51″ E	KZ	OSBU: 25000	OM891915	OM983352	OM983399
Am781	*Allium pallasii*	48°33′43″ N 70°54′16″ E	KZ	OSBU: 24835	OM891916	OM983353	OM983400
Am782	*Allium pallasii*	49°14′1″ N 73°3′58″ E	KZ	OSBU: 24770	OM891917	OM983354	OM983401
Am786	*Allium pallasii*	50°11′54″ N 87°56′31″ E	RU	OSBU: 23556	OM891918	OM983355	OM983402
Am823	*Allium pallasii*	47°48′46″ N 85°27′40″ E	KZ	ALTB: Starikov. 21 May 1990	OM891919	OM983356	OM983403
Am1282	*Allium pallasii*	51°20′11″ N 70°5′25″ E	KZ	OSBU: 24956	OM891920	OM983357	OM983404
Am1283	*Allium pallasii*	49°6′41″ N 66°59′48″ E	KZ	OSBU: 24765	OM891921	OM983358	OM983405
Am1284	*Allium pallasii*	50°15′49″ N 66°59′32″ E	KZ	OSBU:24931	OM891922	OM983359	OM983406
Am1285	*Allium pallasii*	51°41′58″ N 73°25′27″ E	KZ	OSBU: 28157	OM891923	OM983360	OM983407
Am456	*Allium caricifolium*	47°52′6″ N 81°28′34″ E	KZ	ALTB: Kechaikin, 2 June 12	OM891924	OM983361	OM983408
Am491	*Allium caricifolium*	47°55′60″ N 85°7′60″ E	KZ	OSBU: 22298	OM891925	OM983362	OM983409
Am492	*Allium caricifolium*	47°25′53″ N 84°39′11″ E	KZ	OSBU: 22290	OM891926		OM983410
Am493	*Allium caricifolium*	40°31′51″ N 72°47′11″ E	KG	GAT: 0017895	OM891927		
Am494	*Allium caricifolium*	40°16′23″ N 72°37′30″ E	KG	GAT: 0017900	OM891928		
Am553	*Allium caricifolium*	45°50′5″ N 81°19′56″ E	KZ	TK: Goloskokov, 18 June 1959	OM891929	OM983363	OM983411
Am578	*Allium caricifolium*	49°45′59″ N 82°32′9″ E		ALTB: Kechaikin, Tjutjunic, 1 June 2012		OM983364	
Am580	*Allium caricifolium*	49°15′0″ N 81°45′0″ E	KZ	ALTB: Smirnov, Antonjuk, 18 May 2000	OM891930	OM983365	OM983412
Am606	*Allium caricifolium x A.pallasii*	46°59′40″ N 73°26′60″ E	KZ	TK: Pavlov, 28 May 1951	OM891931	OM983366	OM983413
Am608	*Allium caricifolium*	43°51′55″ N 74°8′19″ E	KZ	TK: Pavlov, 11 May 1951	OM891932	OM983367	OM983414
Am686	*Allium caricifolium*	46°55′29″ N 80°36′52″ E	KZ	OSBU: 24335	OM891933	OM983368	OM983415
Am692	*Allium caricifolium*	45°43′55″ N 80°31′33″ E	KZ	OSBU: 24340	OM891934	OM983369	OM983416
Am705	*Allium caricifolium*	45°55′58″ N 80°36′31″ E	KZ	OSBU: 24368	OM891935	OM983370	OM983417
Am708	*Allium caricifolium*	47°14′20″ N 80°43′6″ E	KZ	OSBU: 24370	OM891936	OM983371	OM983418
Am718	*Allium caricifolium*	49°51′16″ N 82°25′26″ E	KZ	OSBU: 24390	OM891937	OM983372	OM983419
Am1192	*Allium caricifolium*	45°39′37″ N 80°34′56″ E	KZ	OSBU: 24354	OM891938	OM983373	OM983420
Am1246	*Allium caricifolium*	43°0′56″ N 74°42′19″ E	KG	OSN:2021-0746-W	OM891939	OM983374	OM983421
Am1274	*Allium caricifolium*	42°36′6″ N 75°53′39″ E	KG	GAT: 3060090	OM891940	OM983375	OM983422
Am1275	*Allium caricifolium*	40°8′29″ N 72°21′47″ E	KG	GAT: 2543277	OM891941	OM983376	OM983423
Am1280	*Allium caricifolium*	42°47′0″ N 74°32′53″ E	KG	FRU: Usulbaev A.K., 20 May 2019	OM891942	OM983377	OM983424
Am1281	*Allium caricifolium*	47°34′42″ N 80°37′47″ E	KZ	OSBU: 24328	OM891943	OM983378	OM983425
Am826	*Allium caesium*	48°3′22″ N 68°28′33″ E	KZ	OSBU: 24961	OM891944		OM983426
Am683	*Allium caeruleum*	47°24′53″ N 80°34′56″ E	KZ	OSBU: 24332	OM891945		
Am473	*Allium delicatulum*	47°52′59″ N 81°28′34″ E	KZ	ALTB: Kechaikin, 2 June 2012	OM891946		
Am573	*Allium delicatulum*	51°44′13″ N 94°28′26″ E	RU	NS: 0014638	OM891947		
Am712	*Allium delicatulum*	47°55′39″ N 82°4′10″ E	KZ	OSBU: 24380	OM891948		OM983427
Am749	*Allium zaissanicum*	48°49′11″ N 83°46′12″ E	KZ	OSBU: 23940	OM891949		
Am1243	*Allium moschatum*	46°47′22″ N 17°18′24″ E	HUN	OSN: 2019-0872-W	OM891950		OM983429
Am1245	*Allium flavum*	48°54′14″ N 21°57′56″ E	SVK	OSN:2004-0826-W	OM891951	OM983379	OM983428
Am1239	*Allium vineale*	48°17′16″ N 16°50′36″ E	A	OSBU: 17147	OM891952		
Am1244	*Allium paniculatum*	49°55′26″ N 42°25′22″ E	RU	OSN: 2018-1203-W	OM891953	OM983380	OM983430
Am881	*Allium praescisum*	52°29′48″ N 61°58′0″ E	KZ	OSBU: 25829	OM891954	OM983381	OM983431
Am630	*Allium amphibolum*	49°17′14″ N 87°43′4″ E	RU	OSBU: 23610			OM983432
Am875	*Allium lineare*	49°6′56″ N 72°40′59″ E	KZ	OSBU: 24823			OM983433
Am848	*Allium strictum*	49°55′58″ N 14°8′31″ E	CZ	FRT 2015/62			OM983434
Am1286	*Allium scabriscapum*	36°2′12″ N 51°12′10″ E	IR	GAT 19116			OM983435
Am1240	*Allium kunthianum*	42°39′52″ N 44°37′0″ E	GEO	OSN: 2020-0819-W			OM983436
Am1241	*Allium oleraceum*	52°21′22″ N 14°10′12″ E	DE	OSN: 2016-0568-W			OM983437
