# Supplementary material for: Allium pallasii and A. caricifolium—Surprisingly Diverse Old Steppe Species, Showing a Clear Geographical Barrier in the Area of Lake Zaysan
Source: Plants (Basel). 2022 May 30;11(11):1465. doi: 10.3390/plants11111465 (PMC9182953; doi:10.3390/plants11111465)
Supplement: Supplementary file 1 [file plants-11-01465-s001.zip › plants-1664682-supplementary.pdf]

Supplements

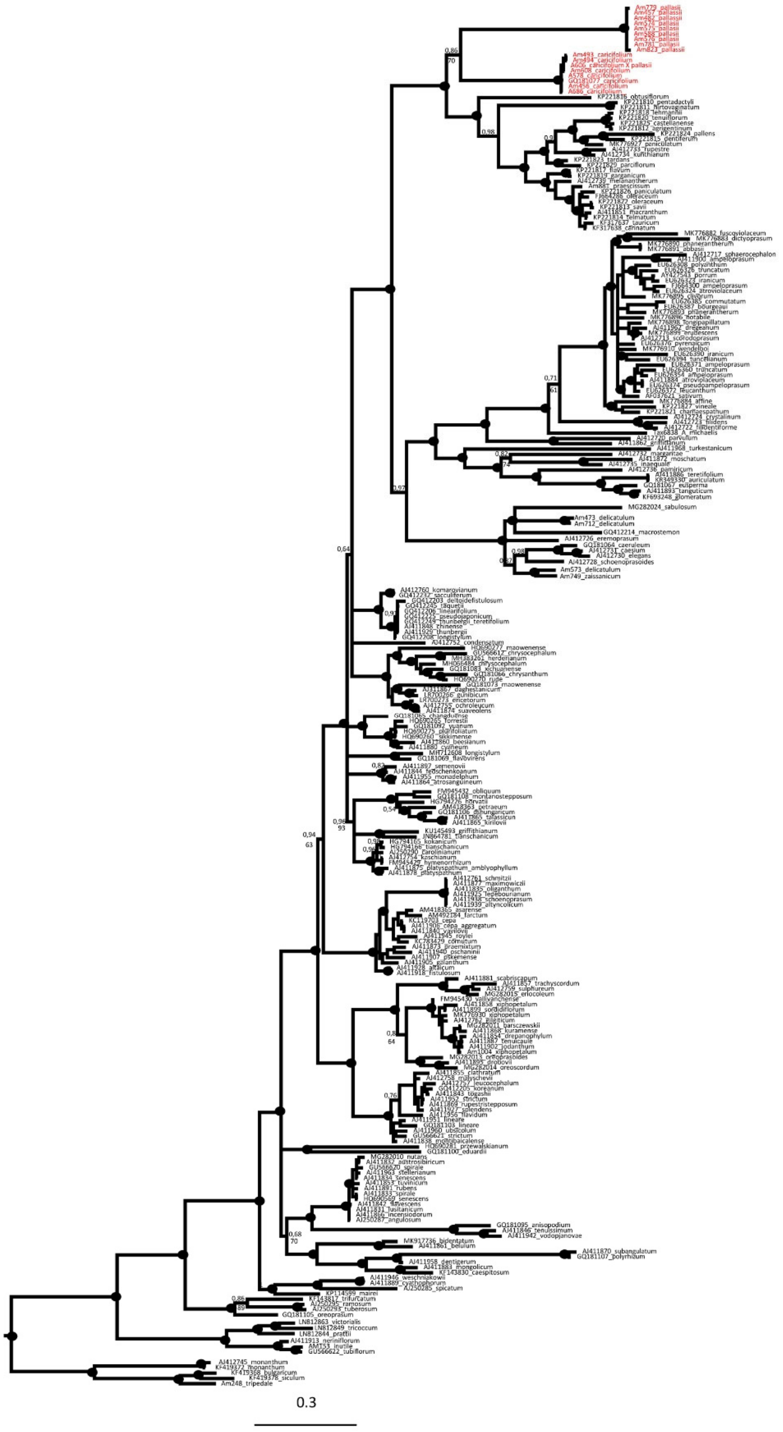

Figure S1. Phylogenetic tree of third evolutionary lineage of the genus *Allium*, based on ITS sequences from NCBI GenBank. Numbers by nodes represent bootstrap support (1000 replicates) and Bayesian probabilities. The joint presence of Bayesian probabilities over 0.98 and bootstrap support over 95% is indicated with a black dot. Origin of samples without GenBank accession number see in Table 1.

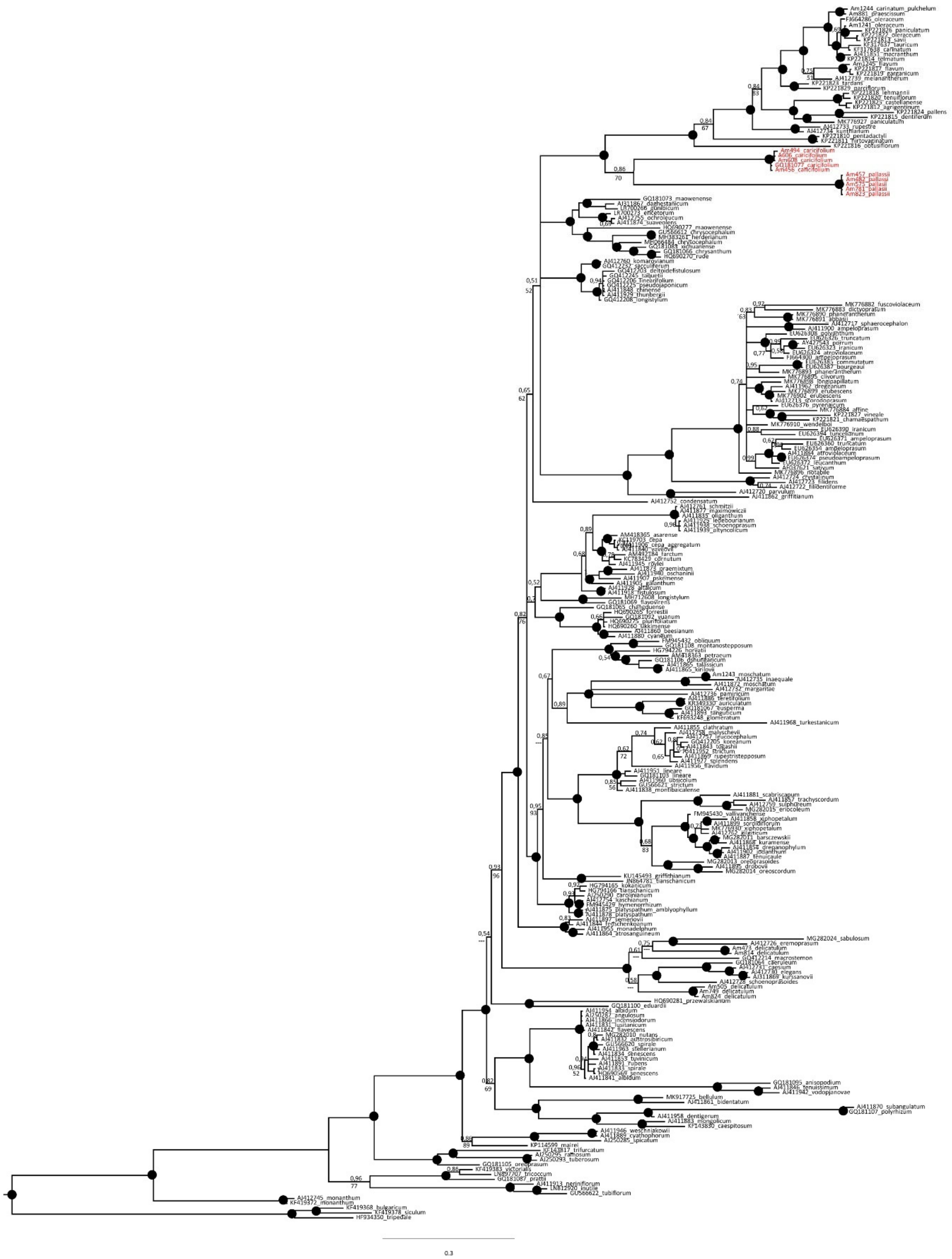

Figure S2. Phylogenetic tree of third evolutionary lineage of the genus *Allium*, based on CP DNA sequences (*trn*L-*rpl*32) from NCBI GenBank. Numbers by nodes represent bootstrap support (1000 replicates) and Bayesian probabilities. The joint presence of Bayesian probabilities over 0.98 and bootstrap support over 95% is indicated with a black dot. Origin of samples without GenBank accession number see in Table 1.

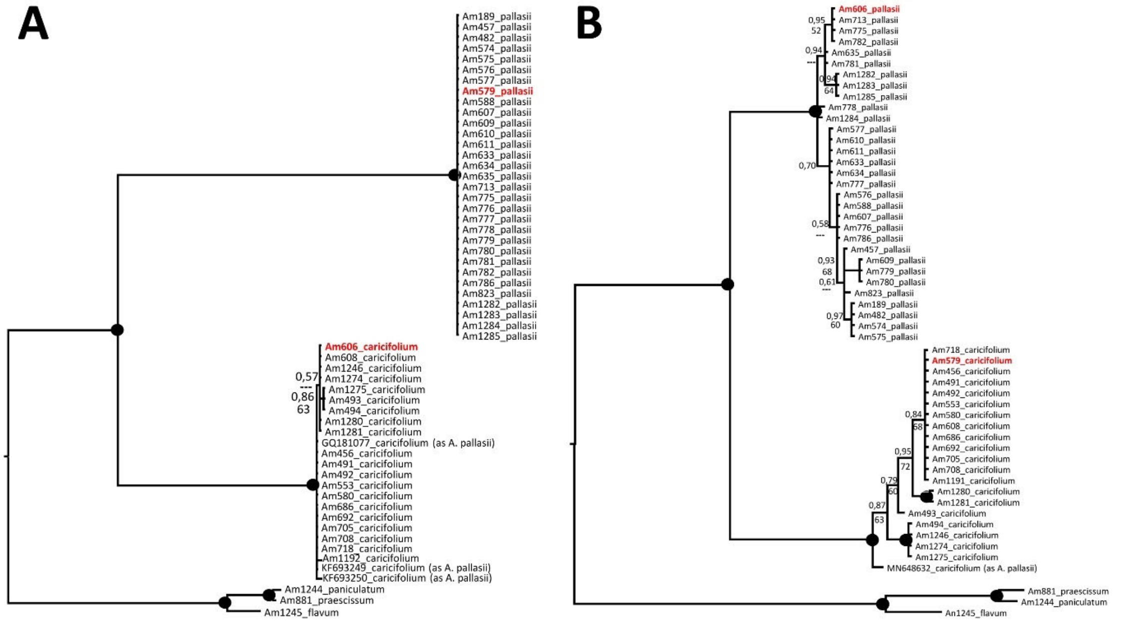

Figure S3. A - Phylogenetic tree of *A*. sect. *Pallasia* accessions**,** based on ITS sequences; B - Phylogenetic tree of *A*. sect. *Pallasia* accessions**,** based on combined two fragments of plastid DNA (trnL-rpl32, trnQ-rps16). Numbers by nodes represent bootstrap support (1000 replicates) and Bayesian probabilities. The joint presence of Bayesian probabilities over 0.98 and bootstrap support over 95% is indicated with a black dot. The origin of Am samples is seen in Table 1.

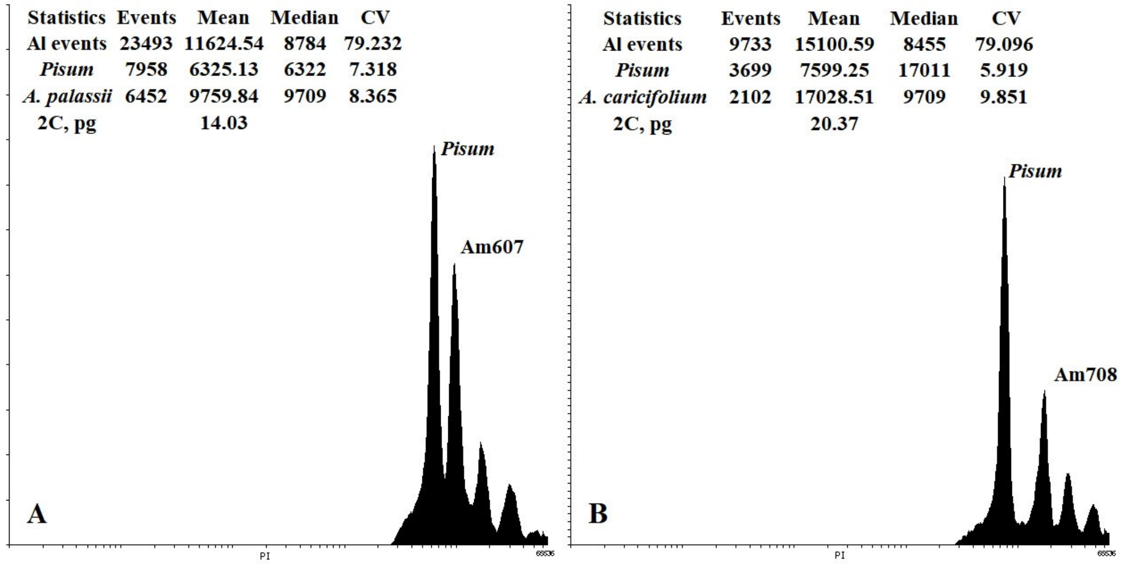

Figure S4. Histograms of relative DNA content were obtained after analysis of nuclei isolated from young leaf tissues of *A. pallasii,* accession Am 607 (A) and *A. caricifolium,* accession Am708 (B). See Photos in Figure 2 D - E. *Pisum sativum* was used as an internal standard.
